# Tail tubular protein A: a dual-function tail protein of *Klebsiella pneumoniae* bacteriophage KP32

**DOI:** 10.1038/s41598-017-02451-3

**Published:** 2017-05-22

**Authors:** Anna Pyra, Ewa Brzozowska, Krzysztof Pawlik, Andrzej Gamian, Miroslawa Dauter, Zbigniew Dauter

**Affiliations:** 1 University of Wroclaw, Faculty of Chemistry, Department of Crystallography, 14 F. Joliot-Curie, Wroclaw, 50383 Poland; 2 Hirszfeld Institute of Immunology and Experimental Therapy, Polish Academy of Sciences, 12 R. Weigl, Wroclaw, 53114 Poland; 3 Leidos Biomedical Research Inc., Basic Research Program, Argonne National Laboratory, Argonne, IL 60439 USA; 4 Synchrotron Radiation Research Section, MCL, National Cancer Institute, Argonne National Laboratory, Argonne, IL 60439 USA

## Abstract

Tail tubular protein A (TTPA) is a structural tail protein of *Klebsiella pneumonia*e bacteriophage KP32, and is responsible for adhering the bacteriophage to host cells. For the first time, we found that TTPA also exhibits lytic activity towards capsular exopolysaccharide (EPS) of the multiresistant clinical strain of *Klebsiella pneumoniae*, PCM2713, and thus should be regarded as a dual-function macromolecule that exhibits both structural and enzymatic actions. Here, we present our crystallographic and enzymatic studies of TTPA. TTPA was crystallized and X-ray diffraction data were collected to a resolution of 1.9 Å. In the crystal, TTPA molecules were found to adopt a tetrameric structure with α-helical domains on one side and β-strands and loops on the other. The novel crystal structure of TTPA resembles those of the bacteriophage T7 tail protein gp11 and gp4 of bacteriophage P22, but TTPA contains an additional antiparallel β-sheet carrying a lectin-like domain that could be responsible for EPS binding. The enzymatic activity of TTPA may reflect the presence of a peptidoglycan hydrolase domain in the α-helical region (amino acid residues 126 to 173). These novel results provide new insights into the enzymatic mechanism through which TTPA acts on polysaccharides.

## Introduction

The strains of *Klebsiella pneumoniae* belong to the *Enterobacteriaceae* family and are widely distributed in the environment^1^. The most common infections caused by these bacteria are pneumonias, such as bronchopneumonia and bronchitis. *Klebsiell*a infections are mostly seen in people with a weakened immune system; these patients have an increased tendency to develop lung abscess, cavitation, empyema, and pleural adhesions. *Klebsiella* infections are characterized by a high death rate of about 50% even with antimicrobial therapy^2–7^. *K*. *pneumoniae*, *S*. *aureus*, *E*. *faecalis*, *P. aeruginosa*, and *E*. *coli* are among the most frequent biofilm-forming microorganisms^8^. A biofilm is an organized structure in which bacterial cells stick to one another on various solid surfaces, forming large aggregates^9^. One of the major biofilm adhesion factors is a bacterial polysaccharide that is secreted outside the cell, and is hence called exopolysaccharide (EPS)^10^. EPS forms a shell around the bacterial cell (called a capsule) that may involve covalent and/or ionic associations with the cell^11^. The EPSs of *K*. *pneumoniae* strains are composed of repeating subunits of three to six sugars in different combinations; there almost 80 such polymers known, most of whose structures mainly contain D-glucuronic acid and some D-galacturonic acid^12, 13^. EPS is critical for the ability of *K*. *pneumoniae* to resist host defense mechanisms, suppress early inflammatory responses, adhere, and form biofilm^14^. Biofilm formation is responsible for over 60% of chronic and recurrent infections^15^, and *Klebsiella* organisms are becoming increasingly resistant to multiple antibiotics. It is an emerging worldwide challenge to develop new-generation medicines against such opportunistic pathogens. A promising alternative for this purpose is the use of bacteriophages (phages), which can destroy bacterial biofilm via depolymerizing enzymes (depolymerases)^4, 16^. The depolymerases bind to the capsular EPS (as a secondary receptor) and degrade the polymer until the phage reaches the cell surface, where it binds to an outer membrane receptor (the primary receptor) and injects nucleic acids to initiate the lytic cycle^17^. Depolymerases are present as part of the phage tail or are secreted outside the cell as a separate enzyme. This process facilitates bacterial infection and produces translucent halos around the clear phage plaques on a bacterial lawn^18^. To date, 160 putative depolymerases have been identified from 143 phages (43 *Myovirida*e, 47 *Siphoviridae*, 37 *Podoviridae*, and 16 unclassified) that infect 24 genera of bacteria^19^. Our latest published search found that the huge majority of phage depolymerases are encoded in the same open reading frame as phage structural proteins (mostly localized on phage tails), and thus should also be considered structural proteins^19^. The phage depolymerases are expected to be diverse, as they act on a wide variety of EPSs.

The KP32 bacteriophage belongs to the *Caudovirales* family and can infect multidrug resistant *K. pneumoniae* isolates^20^. Analysis of the KP32 genome revealed the presence of three tail proteins that are predicted to be involved in the bacterial infection machinery: tail tubular protein A (TTPA), tail tubular protein B (TTPB), and tail fiber protein (TFP). In this paper, we describe the first results relating to these proteins, namely crystallographic and functional studies of TTPA.

## Results and Discussion

**Figure 1:**
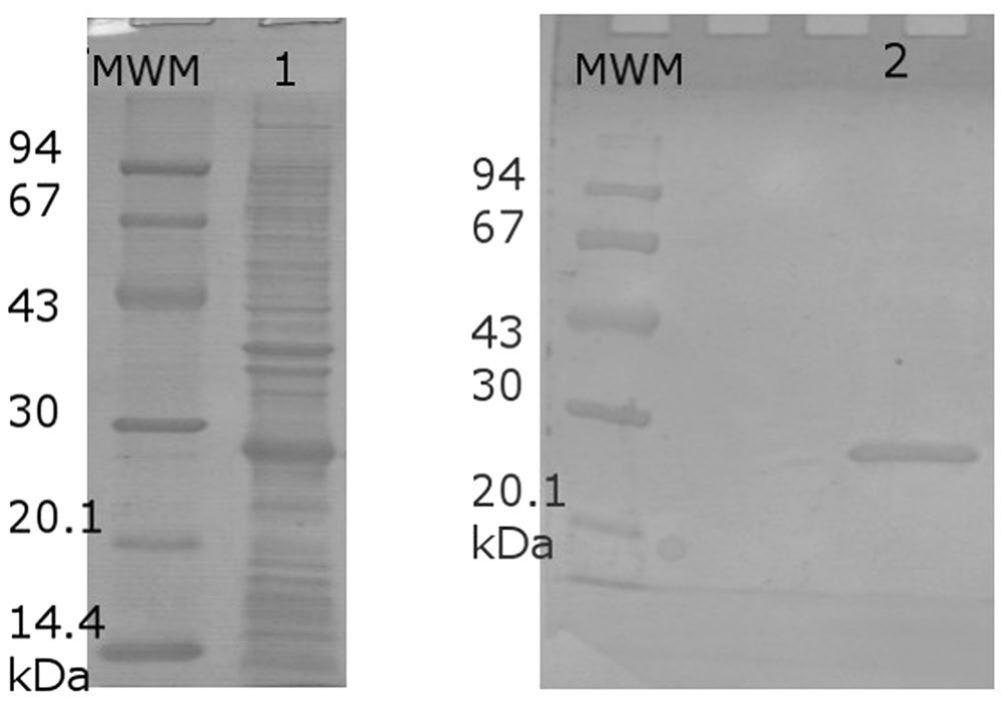
Electrophoretic analysis of TTPA from KP32 phage in 12.5% SDS-PAGE. (MWM) Molecular weight marker (Bio-Rad), (1) TTPA before purification, (2) TTPA after purification by Ni^2+^-affinity chromatography.

The purified TTPA was analyzed by SDS-PAGE (Fig. 1) and matrix-assisted laser-desorption ionization time-of-flight mass spectrometry (MALDI-TOF MS). The apparent molecular weight of TTPA was 21.63 kDa.

**Figure 2:**
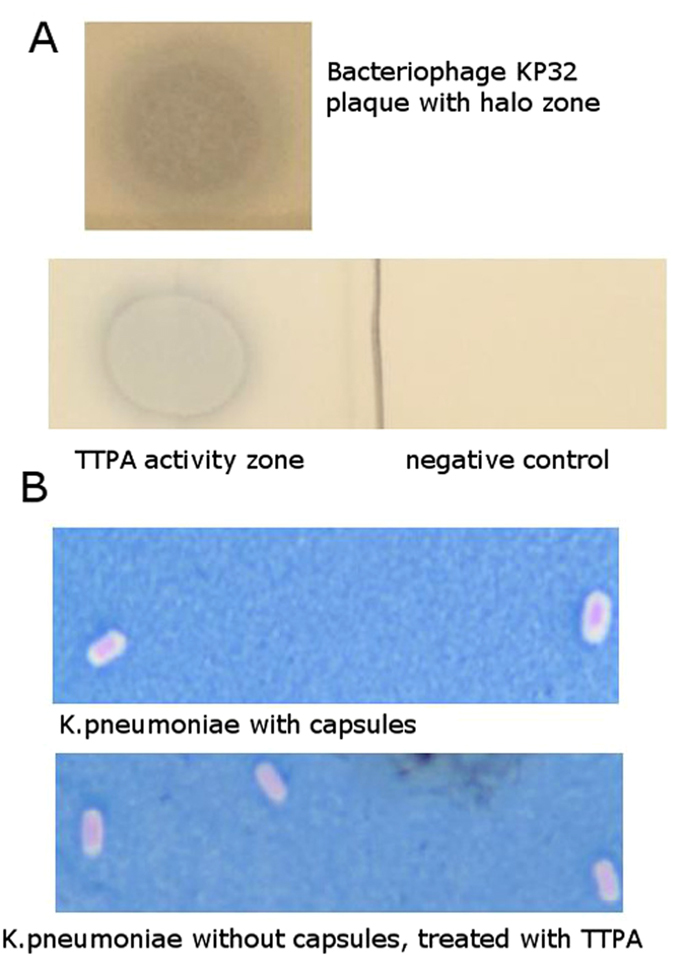
Hydrolytic activity of TTPA. (**A**) The agar layer method. KP32 phage (10 µl 10^−8^ pfu/ml), TTPA (10 µl 0.5 mg/ml), and buffer (negative control) were spotted on a mature bacterial lawn of *Klebsiella pneumoniae* PCM2713 (OD_600_ = 0.2) and the plates were incubated at 37 °C overnight. (**B**) Microscopic analysis of *K. pneumoniae* PCM2713 capsules visualized using Maneval’s stain.

To assess the enzymatic activity of TTPA, we performed agar spot assays using plates with mature bacterial lawns. Our initial studies indicated that TTPA shows the ability to create translucent zones but not clear plaques on the *K. pneumoniae* lawn (Fig. 2A).

Based on the observation that TTPA is active against *K. pneumoniae –* bacteria that are able to produce large amount of slime polysaccharides, we assessed the effect of TTPA on bacterial capsular polysaccharides. Our microscopic studies showed that *K. pneumoniae* cells treated with purified TTPA (10 µl 0.5 mg/ml) were denuded of their capsules, whereas control (untreated) cells were surrounded by capsules (Fig. 2B). As shown in Fig. 2B, the phage-treated capsular area became colorless against the blue background. This strongly suggests that the capsular polysaccharides were destroyed.

It has been shown that *K*. *pneumoniae* phages produce polysaccharide hydrolases that are associated with the tail spike, and that these enzymes remain active even after phage inactivation, forming characteristic halo zones on a spot agar plate assay^21^. The plaques caused by the phage are morphologically different from those caused by their hydrolyzing enzymes under the same test conditions. The active phage both degrades the polysaccharides and infects/destroys bacterial cells, and thus forms clear plaques. Depolymerases, in contrast, destroy the polysaccharides that keep bacteria closely associated within small cell clusters, allowing the non-lysed cells to separate from one another to form translucent (rather than clear) zones^22^. The latter effect was observed when we spotted TTPA on the bacterial lawn.

**Figure 3:**
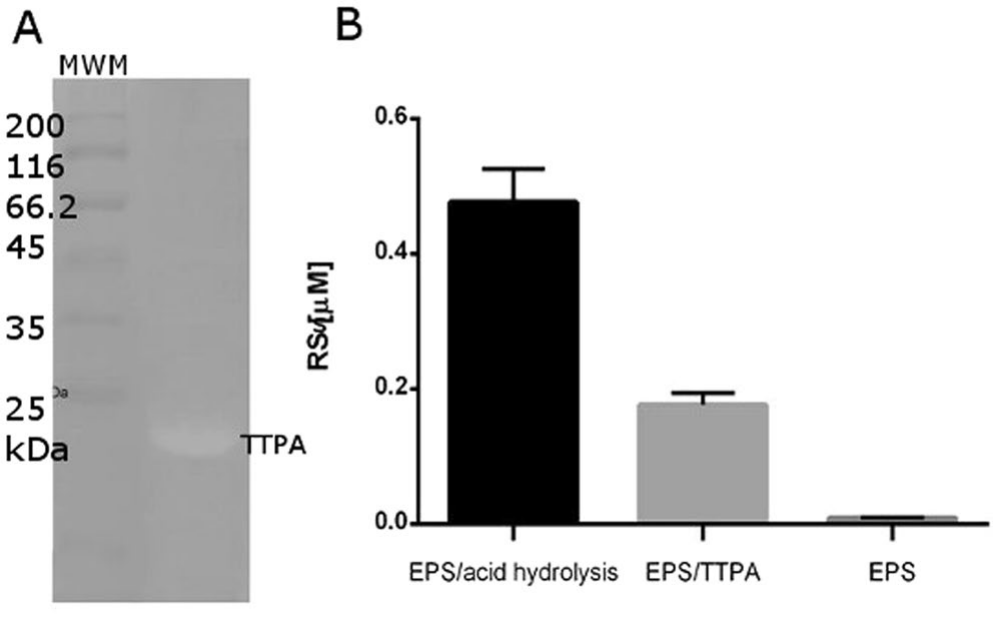
Hydrolytic activity of TTPA against EPS. (**A**) Zymography. Protein samples were resolved by semi-denaturing 12% PAGE on gels embedded with 0.1% substrate (EPS). (MWM) Molecular weight marker (Bio-Rad). (**B**) RSs released from EPS by TTPA versus acid hydrolysis (positive control) or in the absence of TTPA or acid (negative control).

To confirm the enzymatic activity of TTPA, we performed two more experiments using *K. pneumoniae* PCM2713 EPS as a substrate. In the first experiment, we incorporated the EPS into a polyacrylamide gel and used zymography to visualize the enzymatic activity of TTPA. Clearing was observed at 21 kDa, which corresponds to the predicted size of the phage protein (Fig. 3A). In the second experiment, we estimated the amount of reduced sugars (RSs) obtained during the TTPA-mediated depolymerization of EPS. Our results revealed that almost 0.2 µM of RSs was released in the presence of TTPA, whereas no such release was observed in a negative control experiment lacking the enzyme. The amount of RSs released by TTPA was more than 35% of the total RSs obtained when the same amount of EPS was subjected to acid hydrolysis as a positive control (Fig. 3B). We also observed that TTPA exhibited its optimum activity at pH 5.5 (data not shown), which is a characteristic of glycosyl hydrolases^23^.

Hydrolyzing enzymes are mostly associated with the phage tail. Our BLAST amino acid sequence analysis suggests that the composition of the phage KP32 tail is similar to that of T7 bacteriophage and consists of three proteins. These proteins include a fiber protein (gp17) that is responsible for host recognition and the initiation of infection^24^ and TTPA, which is homologous to tail A protein (gp11) of T7 bacteriophage and comes into action after the host bacteria is recognized. TTPA is a structural protein that is located in the bacteriophage tail core; it is necessary for the adhesion of the phage to the bacterial cell wall, which is a prerequisite for host infection^24, 25^. Interestingly, although TTPA is considered a structural protein, it also possesses the depolymerase activity described in this paper. Moreover, *in silico* studies of phage polysaccharide hydrolase-encoding genes have shown that 120 of the 160 different phage-hydrolyzing enzymes are coded in the same open reading frame as structural proteins (tail and fiber proteins), and are thus considered to be structural proteins, not enzymes.

The polysaccharide hydrolyses are very diverse in terms of their molecular weight and specificity. At least 16 different combinations of the catalytic domains of these enzymes have been documented to date^19^. To elucidate the three-dimensional structure and determine the catalytic domains and enzymatic action mode of TTPA, we performed crystallization. The crystallographic data are summarized in Table 1.

**Table 1:** Statistics of diffraction data and structure refinement. Values in parentheses are for the highest-resolution shell.

Data collection	Native	Se-Met
Detector	ADSC Q315	Rayonix 300HS
Space group	P63	P63
Unit cell parameters (Å)		
a = b	138.32	137.39
c	102.93	103.31
Wavelength (Å)	0.9792	0.9763
Resolution limit (Å)	30–1.90 (1.93–1.90)	30–2.30 (2.38–2.30)
Reflections measured	718,659	310,161
Reflections unique	83,194 (4,362)	47,977 (4,888)
Multiplicity	8.2 (6.7)	6.3 (6.0)
Completeness (%)	100.0 (99.8)	100.0 (100.0)
Rmerge (%)	10.7 (86.3)	9.3 (85.5)
Rmeas (%)	11.3 (93.0)	10.1 (94.0)
Average I/σ(I)	20.1 (1.9)	20.2 (2.6)
Refinement		
Resolution (Å)	30–1.90	
R factor (%)	18.31	
Work reflections	81,538	
R_free_ (%)	22.96	
Free reflections	1,650	
R.m.s.d. bond length (Å)	0.021	
R.m.s.d. bond angle (°)	1.957	
Residues in favored conformation (%)	94.14	
Residues in allowed conformation (%)	3.95	
PDB code	5MU4	

**Figure 4:**
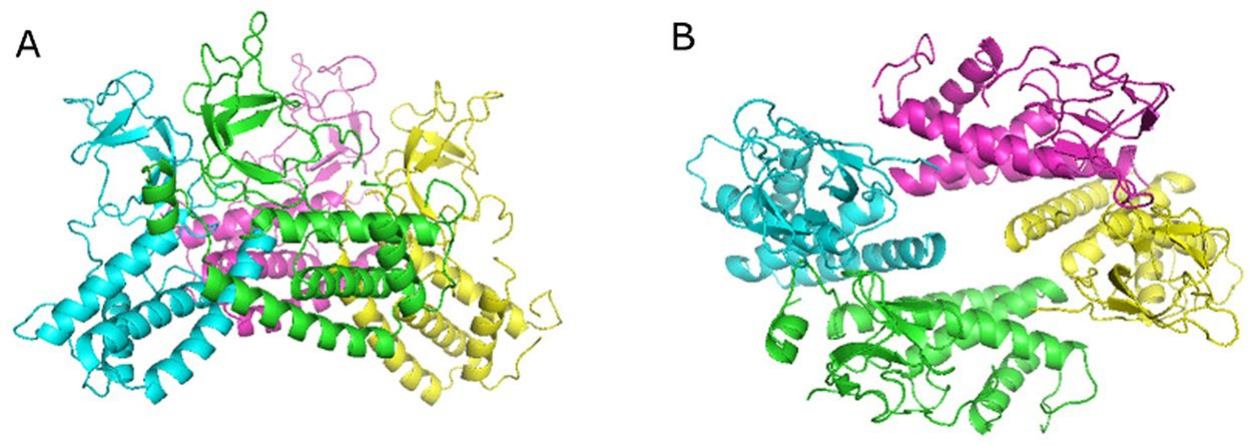
The crystal structure of the TTPA tetramer. Chain A is presented in green, chain B in cyan, chain C in magenta, and chain D in yellow. (**A**) The view along the c-axis. (**B**) The view along the a-axis. The images were prepared in PyMol^51^.

The crystal structure of TTPA distinguished this enzyme from other closely related homologous proteins, in that the crystal TTPA molecules were found to adopt a tetrameric structure with a compact α-helical domain on the one side and β-strands and loops on the other (Fig. 4).

**Figure 5:**
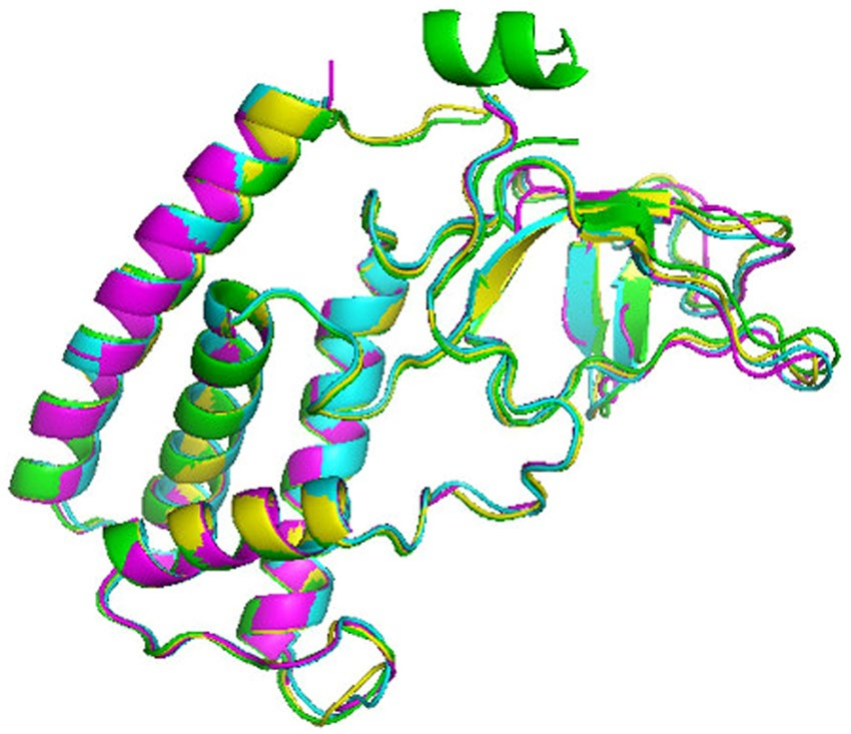
TTPA monomers superimposed onto each other. Chain A is presented in green, chain B in cyan, chain C in magenta, and chain D in yellow. The image was prepared in PyMol^51^.

All four monomers are folded almost identically (Fig. 5). The α-helical domains consist of four helices and five β-strands that form two antiparallel β-sheets. The TTPA molecule consists of 192 amino acid residues; in the electron density map, however, all of the chains have an additional nine expression-plasmid-derived amino acids at the N-terminus (numbered −8 to 0). In addition, not all of the main chain residues are visible in the electron-density map. Chain A is the longest and is entirely interpreted in the electron density map, with the exception of residues 181–184. The electron density maps are interpretable for amino acid residues −8 to 175 of chain B and −8 to 176 for chains C and D. Thus, about 15 residues at the C-termini of chains B, C, and D are not seen in the crystal structure. In chain A, most of the C-terminal part (except for residues 181–184) is visible and forms a well-defined α-helix. There is no interpretable electron density for the side chains of the following residues: Q4, E34, D35, and R49 in chain A; E34, D35, and S74 in chain B; E34, D35, R95, and Y100 in chain C; and E34, D35, and R95 in chain D. Our PROCHECK analysis found that 14 residues are in the disallowed regions of the Ramachandran plot^26^; these include glycines and residues that are localized on the external loops and directed toward the environment, where the electron-density map is poorly defined. The models of the four side chains adopt multiple conformations. The solvent water molecules were classified on the basis of electron density and B factors into 885 fully occupied and 10 half-occupied sites. The structure was refined at a resolution of 1.9 Å to an R factor of 18.3% and an R free of 22.8% in an isotropic mode.

**Figure 6:**
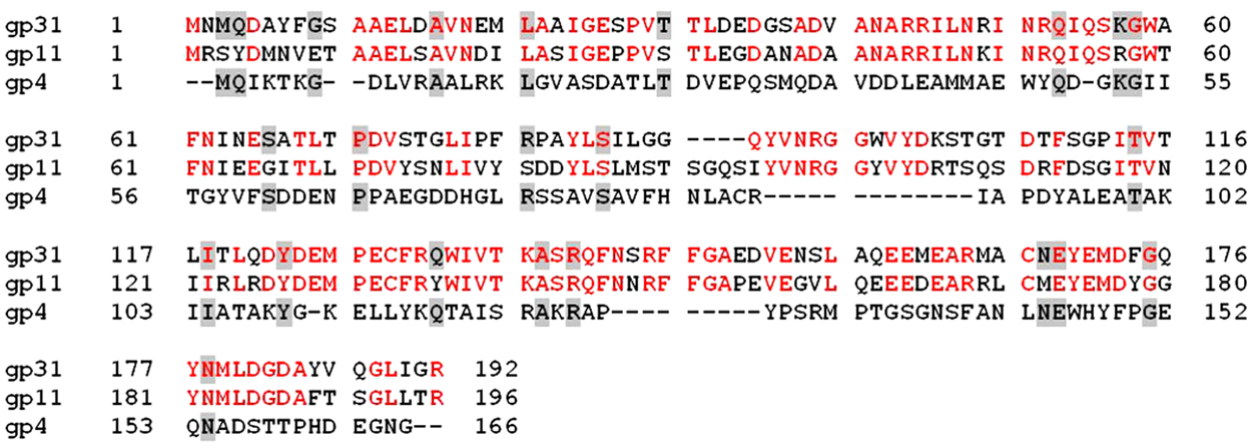
Sequence alignment of TTPA, gp11 from the tail of bacteriophage T7 (PDB code 3j4b), and gp4 from bacteriophage P22 (PDB code 1vt0). Identities between TTPA and gp11 or gp4 are shown in red and shadowed, respectively.

**Figure 7:**
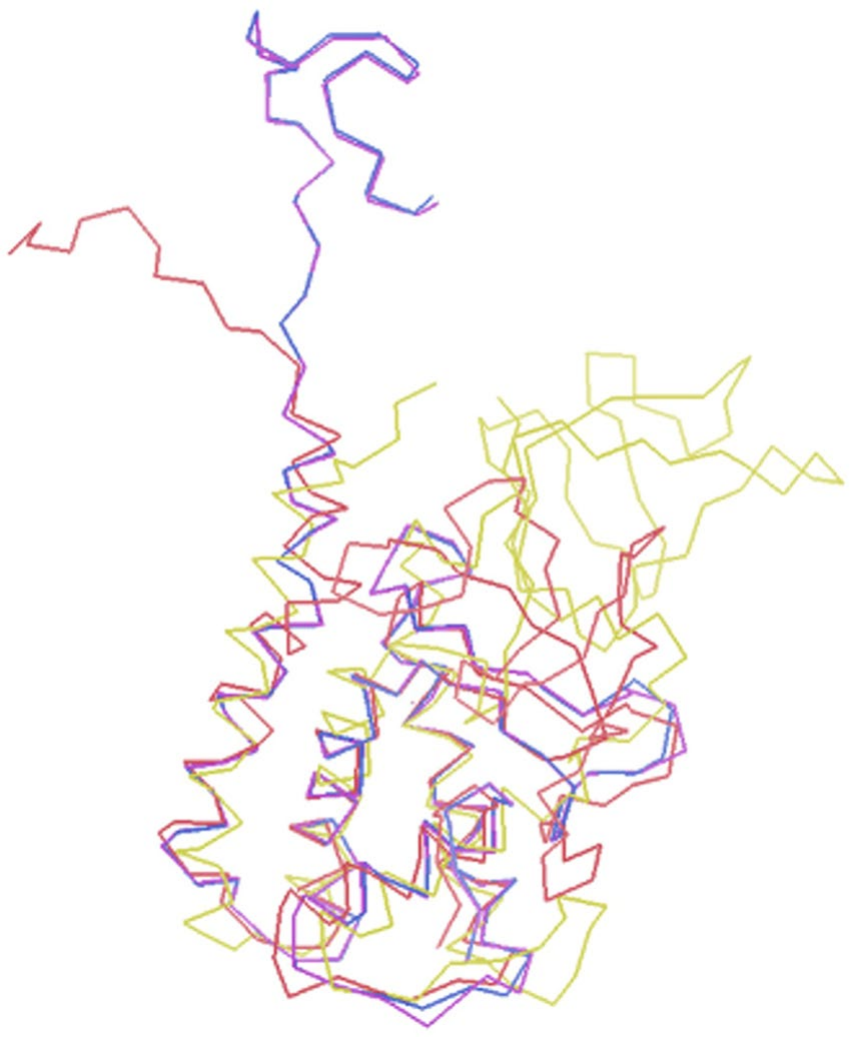
The structures of the TTPA monomer (in yellow), gp11 from bacteriophage T7 (in red; PDB code 3j4b; cryoEM), gp4 from bacteriophage P22 (in dark blue; PDB code 1vt0; X-ray), and gp4 from bacteriophage P22 (in magenta; PDB code 3lj4; cryoEM) superimposed onto each other. The images were prepared in Coot^49^.

The helical domain of the TTPA molecule is very similar to those of bacteriophage T7 tail protein gp11 (PDB code 3j4b, cryoEM) and gp4 of bacteriophage P22 (PDB code 1vt0, X-ray; PBD code 3lj4, cryoEM)^27–29^, which is classified as a DNA stability protein/peptidoglycan hydrolase. Figure 6 presents the aligned sequences of TTPA, gp11, and gp4, and Fig. 7 shows these molecules superimposed on each other.

The root mean square (r.m.s.) difference between TTPA and gp11 calculated for 98 structurally corresponding Cα atoms was 2.551 Å and that between TTPA and gp4 calculated for 99 structurally corresponding Cα atoms was 2.620 Å. Our analysis of the primary structures of TTPA, gp11, and gp4 showed that gp11 and gp4 are 64% and 6% identical to TTPA, respectively. Thus, we thought that gp11 could serve as potential model for solving the TTPA structure by molecular replacement. However, our attempts were not successful. This failure appears to be explained by our crystallographic studies. As mentioned above, the helical domain of TTPA is very similar to those of gp11 and gp4. As in gp11 and gp4, the TTPA monomer has three helices packed together to form a small hydrophobic core, and the fourth and longest helix (that at the C-terminus) is located on the outer surface of the monomer. However, the TTPA molecule has an additional domain consisting of two antiparallel β-sheets and loops, which lacks in gp11 and gp4. Moreover, the crystallized TTPA molecules adopt a tetrameric structure in which the helical domains are directed toward one another to generate the hydrophobic core, while the loop/β-sheet regions are directed to the outside; thus, there is relatively limited contact between monomers. In the crystal structure of gp4, in contrast, the monomers adopt a dodecameric ring structure in which the contacts between monomers are generated only by helices. According to electron microscopic analysis, gp11 also forms dodecamers.

Next, we set out to localize the binding sites of TTPA. We performed Phyre2 server analysis^30^ and compared our results with those obtained from our crystallographic studies of TTPA. Our Phyre2 server analysis (confidence 67.3% and identity 21%) showed that the antiparallel β-sheet region (residues 57–99) of TTPA may contain a lectin-like domain; as such domains interact with oligosaccharides, this finding suggests that TTPA could display glucanase activity. These results agreed with the crystal structure of TTPA, wherein residues 57–99 adopt an antiparallel β-sheet that is absent from gp11 and gp4.

A previous report found that although enzymes from different glycosidase families can have little or no sequence homology, their catalytic domains are typically located at the C-terminus in a region that forms a β-strand motif ^31^. The tail phage proteins represent a class of carbohydrate-binding proteins that differ from lectins and lack the carbohydrate-binding modules that are integral to the known glycoside hydrolases^32^. However, our present work shows that, as in other phage tail proteins, lectin-like contacts appear to be responsible for the interactions between TTPA and carbohydrates.

We also sought to identify the site responsible for the EPS depolymerase activity of TTPA. For this purpose, we assessed the 3D structure homology rather than sequence homology because the 3D structures of the proteins in question are more highly conserved than their sequences^23^. As mentioned above, the tertiary structures of TTPA and gp4 are similar, even though the proteins share only 6% sequence homology. Consistent with the classification of gp4 as a peptidoglycan hydrolase, our analysis using the HHpred Bioinformatics server^33^ showed that TTPA contains a hydrolytic domain within amino acid residues 126 to 173 (score 24.68). In most cases, the hydrolysis of the glycosidic bond takes place via a general acid catalysis that requires two critical residues: a proton donor and a nucleophile/base. Two major mechanisms have been proposed for the enzymatic hydrolysis of glycosidic bonds: retention and invertion^23^. In most of the glycosyl hydrolases studied to date, only aspartate and/or glutamate residues have been found to perform catalysis. In general, the carbonyl oxygen of the substrate acts as a nucleophile, with assistance from a carboxylate Asp/Glu that acts to deprotonate the N-acetamido nitrogen during oxazolinium ion formation/breakdown. A second catalytic acid residue acts as a general acid/base to: 1) protonate the glycosidic oxygen to assist in the departure of the aglycon; and 2) deprotonate the nucleophilic water molecule during the hydrolysis of the oxazolinium ion intermediate^34^. In most glycosyl hydrolase families, the two catalytic carboxylates are found in a D-X-E motif; in the other families, the carboxylates may be adjacent, such as in a D-D motif^34^. We therefore propose that the D121-X-D123 and D151-X-E153 residues of TTPA may be good candidate catalytic residues. Further mutant studies of TTPA will be required to confirm these suggestions.

Until now, TTPA was considered to be solely a bacteriophage tail structure protein responsible for the adhesion of the bacteriophage to a host cell. However, our present biological studies collectively show that TTPA also exhibits a depolymerase activity. Similar activities have been observed in other bacteriophage proteins^35^, such as gp4, but these proteins are completely different in their primary structures. The structural and enzymatic components of TTPA make it a very interesting dual-function protein.

## Conclusions

The growing need to treat infections caused by multiresistant species of *Klebsiella pneumoniae* means that researchers must seek alternative methods to effectively eradicate these bacteria. One potential strategy is phagotherapy using lytic bacteriophages, either as whole cells or by applying structural elements of their virions. The use of structural macromolecules, such as proteins, to prevent or remove biofilm formation could lead to the development of stable synthetic drugs that may be mass produced. This could be of interest for the pharmaceutical industry, in that it could support the discovery of new antibiotics that can be applied in hospitals.

Here, we determined the crystal structure of TTPA, which we also report herein for the first time as a dual-function protein. As a bacteriophage tail structural protein, TTPA is involved in the adhesion of the phage to a bacterial cell. However, it also displays a newly identified high-level activity as a bacteriophage hydrolase that acts against pathogenic bacterial exopolysaccharide. Our crystallographic results show that although TTPA has a helical domain similar to the known bacteriophage tail proteins, gp4 and gp11, it contains additional structural elements that distinguish its 3D structure from all others found in PDB. The sequence of TTPA is very similar to that of the bacteriophage T7 tail protein, gp11; unlike gp11, however, TTPA can depolymerize bacterial exopolysaccharides. It is worth noting that saccharide diversity means that there is a great variety in the enzymes that hydrolyze glycosidic bonds. To understand their mechanisms of action, it seems more useful to examine 3D structure homology rather than sequence homology. Structural studies such as this one therefore add critical information to the field. Moreover, our present results constitute an initial step toward determining the enzymatic mode of action of TTPA.

## Materials and Methods

### Gene cloning, overexpression, and protein purification

Bacteriophage gene gp31, which encodes TTPA, was amplified by polymerase chain reaction (PCR) using Taq polymerase (Fermentas) and the following primers: TTPA_FW, GGATCCCATATGAACATGCAAGATGCTTAC and TTPA_RV, GAATTCAAAGCTTACGACCGATGAGACCCT. The obtained PCR product was cloned into the pGEM T-easy vector (T-vector, Promega) using T4 ligase. The construct was transformed into *E. coli* DH5*α* cells using the heat-shock method, and then sequenced. The correct sequences were cloned into the pET28a expression vector (Promega). The obtained plasmids were transformed into *E. coli* BL21(DE3)plysS competent cells, which were grown to an OD_600_ of 0.8 and then induced overnight with 0.05 mM IPTG at 9 °C in Luria-Bertani (LB) medium. The cells were harvested by centrifugation and lysed by sonication in 50 mM Tris/HCl buffer, pH 8.0, containing 0.2 M NaCl and 5% glycerol (buffer A), and supplemented with a protease inhibitor cocktail tablet. After sonication, the cell debris was removed by centrifugation. The supernatant was mixed with Ni^2+^-agarose beads, equilibrated with buffer A, and incubated for 1 hour at 37 °C on a rotary shaker. Unbound proteins were removed by several washes with buffer A. The purified TTPA was eluted with buffer A containing 250 mM imidazole. The imidazole was removed, and the protein was concentrated using a 3-kDa cutoff centrifugal filter (Millipore). The final yield was 11 mg/ml, as measured by the BCA method described by Smith *et al*.^36^ or by the Bradford assay^37^. To obtain the selenomethionyl (Se-Met) derivative of TTPA, the bacteria were grown in selenium-enriched medium. The purity of the obtained TTPA was analyzed by 12.5% SDS-PAGE according to the method of Laemmli *et al*.^38^.

### *K. pneumoniae* strain and hydrolytic activity assay using agar overlay

*K. pneumoniae* PCM2713 was obtained from the Polish Collection of Microorganisms (PCM) of the Institute of Immunology and Experimental Therapy, Polish Academy of Sciences (Wroclaw, Poland). The strain was stored at −80 °C and cultivated in LB medium (Difco) at 37 °C with shaking. The hydrolytic activity of TTPA was determined using the spot assay described by Adams and Park^39^. Overnight cultures of bacteria were diluted to OD_600_ = 0.2 and pipetted onto agar plates, 10 µl of protein (0.5 mg/ml) was spotted on the bacterial lawn, and the plates were incubated at 37 °C overnight. The nutrient agar (pH 7.2) was composed of beef extract (10 g), peptone (10 g), NaCl (5 g), and agar (20 g) dissolved in 1000 ml of water, and was sterilized at 121 °C for 20 minutes.

### Zymography

Purified TTPA (2 μg) was tested against *K. pneumoniae* EPS using zymography. PAGE was performed as described previously^40^ under semi-denaturing conditions with 0.1% of substrate (EPS) embedded in the gel.

### EPS preparation and depolymerase activity measurement

The capsular EPS of *K. pneumoniae* PCM2713 was extracted from the freeze-dried bacterial mass using 10% trichloroacetic acid (TCA) according to the method described by Gorska-Frączek *et al*.^41^. The freeze-dried preparation of crude EPS (20 mg) was dissolved in 1 ml of buffer (50 mM Tris-HCl, pH 7.5, 10 mM MgCl2) and treated with DNase (210 μg; Sigma) and RNase (210 μg; Sigma) at 37 °C for 6 h. This step was followed by an overnight hydrolysis with *Streptomyces griseus* protease (447 μg, 37 °C; Sigma). Finally, the preparation was dialyzed against water at 4 °C for 24 h. To test for depolymerization activity, 0.1 ml of EPS (1 mg/ml) was mixed with 0.1 ml of TTPA (0.5 mg/ml). The reaction mixture (pH 5.5) was incubated for 2 h at 25 °C on a rotary shaker. The reducing sugar (RS) concentration was determined with glucose as a standard, according to the Nelson-Somogoyi method^42^. As a positive control, we used the RS obtained following acidic hydrolysis of EPS (1 mg/ml) with 6 M HCl at 115 °C in an autoclave for 1 h^43^. The negative control was run with EPS but no TTPA.

### Bacterial capsule staining

*K. pneumoniae* cells were cultured on agar plates and treated with or without 10 μl of TTPA (0.5 mg/ml) for 1 h. The capsules were stained using Maneval’s stain, which contained phenol (5%), glacial acetic acid (20%), FeCl_3_ (30%), and Congo red (1%)^44^. Briefly, a drop of bacterial cell suspension was deposited on a clean, grease-free slide, smeared with a drop of the Congo red solution, dried, and then flooded with Maneval’s stain for 2 minutes. The excess stain was discarded and the preparation was observed under a microscope.

### Crystallization and data collection

The native TTPA protein was preliminarily crystallized by the sitting-drop method using robotic screens. Small needle-shaped crystals of 0.01 × 0.01 × 0.1 mm in size appeared after about two weeks. The crystals were obtained by mixing the protein solution (11 mg/ml, 20 mM Tris-HCl, pH 8.2, 200 mM NaCl, 5% glycerol) with a well solution (35% Tacsimate, pH 7.0) at a volume ratio of 1:1. After a preliminary robotic screening and subsequent optimization of the crystallization conditions, we grew the Se-Met crystals of TTPA by mixing the protein solution (16 mg/ml, 20 mM Tris-HCl, pH 8.2, 200 mM NaCl, 5% glycerol) with a good solution (1.1 M sodium malonate, pH 7.0, 0.1 M HEPES, pH 7.0, 0.5% v/v Jeffamine ED-2001, pH 7.0) at a 2:1 ratio. Prior to X-ray diffraction measurements, both native and Se-Met crystals were soaked in the reservoir solution supplemented with 25% glycerol as a cryoprotectant. The X-ray diffraction data of the native and Se-Met TTPA crystals were collected on the SER-CAT beamline 22ID at the APS (Argonne National laboratory, USA) using the CCD detector, ADSC Q315 (for native data), and a Rayonix 300HS detector (for Se-Met data).

### Crystal structure determination

We used the amino acid sequence of TTPA to search all structures deposited in the Protein Data Bank (PDB) using the molecular replacement method, but found no potential structural model that could be used to solve the crystal structure of TTPA. Thus, we obtained the Se-Met derivative crystals of TTPA and used them to solve the crystal structure with the SHELXD/E programs^45^. The protein polypeptide chain was built into an electron density map using the wARP/ARP program^46^. Refinement and water selection were performed with REFMAC5^47^ in the CCP4i package^48, 49^. Real-space model corrections were performed with Coot^50^. The structure quality was validated with PROCHECK^51^. The figures representing the crystal structures were prepared in Coot^50^ or PyMol^52^. The coordinates and structural factors were deposited in PDB and assigned the accession code, 5MU4.
